# The importance of past rifting in large igneous province development

**DOI:** 10.1038/s41586-025-09668-7

**Published:** 2025-11-05

**Authors:** R. Kounoudis, I. D. Bastow, C. J. Ebinger, S. Goes, P. Zhou, M. Musila, C. S. Ogden, A. Ayele

**Affiliations:** 1 Department of Earth Sciences, University of Oxford, Oxford, UK; 2 Department of Earth Science and Engineering, Imperial College London, London, UK; 3 Department of Earth and Environmental Sciences, Tulane University, New Orleans, LA USA; 4 Institute of Geophysics, Space Science and Astronomy, Addis Ababa University, Addis Ababa, Ethiopia

**Keywords:** Tectonics, Seismology, Geophysics, Geodynamics, Volcanology

## Abstract

Lithospheric thin zones, such as recently failed rifts, are generally assumed to be weak spots where magmatism and deformation can concentrate during rifting and large igneous province development^1–3^. Yet, the Turkana Depression in East Africa, the site of the failed 66-million-year-old Anza Rift, did not experience the widespread flood magmatism seen on the adjacent Ethiopian Plateau, despite being a lithospheric thin spot when the region encountered hot plume material around 45 million years ago^4^. Here we jointly invert surface-wave and receiver function data to constrain crustal and upper-mantle seismic structure below the Depression to evaluate lithospheric thermo-mechanical modification. Evidence for thick lower crustal intrusions, ubiquitous below the uplifted Ethiopian Plateau^5,6^, is comparatively lacking below the Depression’s failed Anza Rift system, which ongoing East African rifting is circumnavigating, not exploiting. The mantle lithosphere below the Depression has also retained its cool, fast-wavespeed ‘lid’ character, contrasting the Ethiopian Plateau. Volatile depletion during failed Anza rifting probably rendered the thinned lithosphere refractory without later rejuvenation. Subsequent rifting and magmatism thus initiated away from the still-thin Anza Rift, in regions where fertile lithosphere enabled melting and the sufficient lowering of plate yield strength. Areas of thinned lithosphere are thus not necessarily persistent weak zones where significant extension and magmatic provinces will develop.

## Abstract (web summary)

Seismic data from the Turkana Depression in East Africa show that areas of thinned lithosphere are not necessarily persistent weak zones where extension and magmatic provinces will develop.

## Main

Ancient continental flood-basalt provinces and magmatic rifted margins mark some of Earth’s most voluminous magmatic events^7^. Often associated with the presence of mantle plumes, their development is expected to alter plate thickness and thermo-mechanical structure significantly^8^. However, as post-large igneous province (LIP) cooling has re-defined the thickness and structure of the plates, the extent of this modification, including its development through time, can only be inferred from theoretical models, or from the geological record preserved at ancient LIPs. Consequently, the influence of pre-existing variations in plate thickness and volatile content during LIP formation and subsequent rift development remain poorly understood. Plume magmatism is commonly expected to exploit zones of thinned lithosphere^1–3^. These thin zones are also often assumed to be rheologically weaker, promoting deformation and magmatism by enabling strain localization and adiabatic decompression of underlying plume material that subsequently rises through the weakened lithosphere^9,10^.

**Fig. 1:**
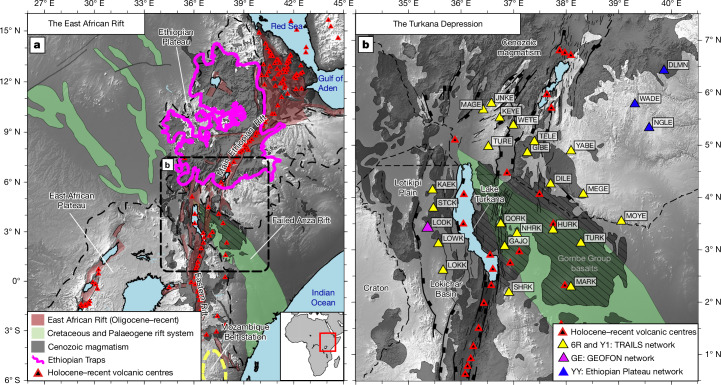
Rifting and magmatism in East Africa. **a**, Mesozoic (Anza) and Cenozoic (East African) rift systems. The dashed box shows the Turkana Depression’s Oligocene–recent and Cretaceous–Palaeogene rift basins and faults^17,50^. The red triangles are Holocene–recent eruptive centres. The shaded grey regions and the magenta outline show Cenozoic magmatism and the Ethiopian flood-basalt province, respectively^19^. The yellow dashed line indicates the location of a reference station within the Mozambique Belt used for comparison^26^. **b**, Seismograph stations in the Turkana Depression. TRAILS, Turkana Rift Arrays Investigating Lithospheric Structure; GEOFON, GEOFOrschungsNetz Seismic Network.

**Fig. 2:**
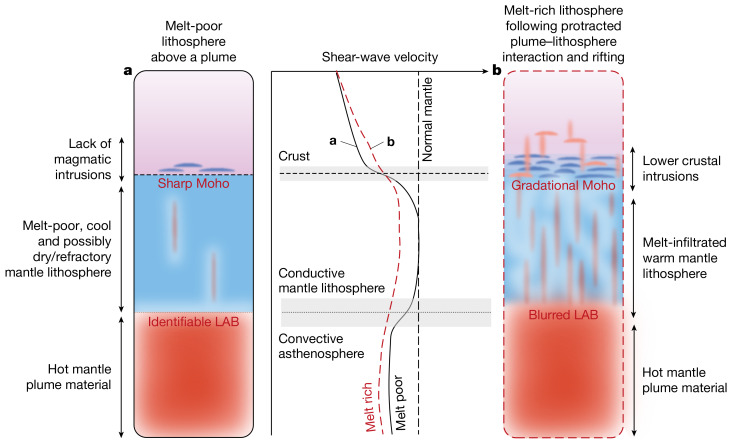
How heating and magmatic intrusions alter lithospheric structure. **a**,**b**, Schematic contrasting regions of minor (**a**) and major (**b**) magmatic modification, including their crustal and uppermost-mantle seismological characteristics.

East Africa (Fig. 1a) offers a unique opportunity to examine plate modification during plume–lithosphere interaction as it is host to the world’s youngest continental flood-basalt province—the Ethiopian Traps—whose main phase saw 1–2 km of flood basalts erupt in the Oligocene epoch (31–29 million years ago (Ma)), concomitant, and spatially coincident, with the development of broad-scale Ethiopian Plateau uplift and onset of extension^11,12^. Below the Ethiopian Plateau, voluminous intrusive magmatism is observed as an 8–12 km-thick lower-crustal-intrusion layer^5^. Deeper still, the lithospheric mantle has seismic wavespeeds that are barely faster than those of the convecting slow-wavespeed asthenosphere below^6,13^ (Fig. 2b), consistent with the view that the plateau lithosphere has been heavily modified by heating and magma intrusion in the Cenozoic era.

Immediately to the south of the flood-basalt-capped Ethiopian Plateau lies the Turkana Depression, which also overlies plume-influenced mantle^14–16^. The Depression was the site of the failed Anza Rift system in the Mesozoic era (primarily Late Cretaceous; about 100–66 Ma)^17^, which stretched the crust by a factor of ≤2.11 (ref. ^18^). This rendered parts of the Depression a lithospheric thin spot during LIP development (Fig. 1a), as the mantle lithosphere would have only partially thermally equilibrated before plume arrival in the Eocene epoch ≤20 Myr later: models estimate 30–60 Myr must elapse before the net effect of permanently thinned crust and re-thickening mantle lithosphere sufficiently restores plate strength to suppress further extension and magmatism^10^. Yet, voluminous early Eocene flood-basalt magmatism (45–32 Ma)^16,19^ and subsequent Oligocene–recent East African rifting^20,21^ only developed west of Lake Turkana near the craton edge (Fig. 1b). East of the lake, where the main Anza depocentres lie^20^, Eocene-age flood-basalt volcanism and Miocene–recent rifting and magmatism are comparatively lacking; Pliocene–recent magmatism comprises mainly isolated shield volcanoes^22^.

Analysis of broadband seismic data across the Depression reveals bulk-crustal *V*_P_/*V*_S_ (compressional-to-shear wavespeed) ratios that are markedly lower (about 1.74)^18^ than below the plateau (*V*_P_/*V*_S_  > 1.8)^23^, implying that the relative lack of widespread mafic magmatism compared with the north persists at crustal depths. These observations collectively challenge the notion that thin lithosphere (for example, the Anza Rift) is inherently weak and enhances magmatic volumes through extension-related melt generation and drainage of plume material^2,9^; a hypothesis often invoked to explain notable along-strike variations in many magmatic rifted margins worldwide^24,25^. The Depression thus offers a unique opportunity to examine plume–lithosphere interactions and rift development in a region of previously rifted and still-thin lithosphere. Key to understanding better the extent to which Eocene–recent magmatism has impacted the Depression’s failed rift zones is an improved knowledge of its lithospheric seismic and thermal structure.

Utilizing data from seismograph deployments in the Turkana Depression and surrounding regions (Fig. 1b), we constrain absolute one-dimensional shear-wave velocities below a network of 38 seismograph stations via joint inversion^26^ of fundamental-mode Rayleigh-wave group velocities (4–100-second period)^27,28^ and P-to-S receiver functions^18^. We pay close attention to the Moho, whose architecture will vary according to the volume of lower crustal intrusions. The lithosphere–asthenosphere system is also a major focus—specifically, whether or not a fast-wavespeed mantle lithospheric lid is readily discernible from hot, plume-affected asthenosphere (Fig. 2). Thermodynamic conversion of shear velocities to temperatures permits a thermal, as well as seismological, means of defining the transition from conductive to convective mantle and allows us to assess variations in mantle potential temperature across the region. Our study demonstrates that lithospheric heterogeneity from previous tectonic events (for example, past rifting)—beyond variations in plate thickness—strongly influences where rifting and LIPs subsequently develop, and may be akin to processes that once shaped ancient continental LIPs and magmatic rifted margins globally.

## Plate wavespeed and thermal structure

**Fig. 3:**
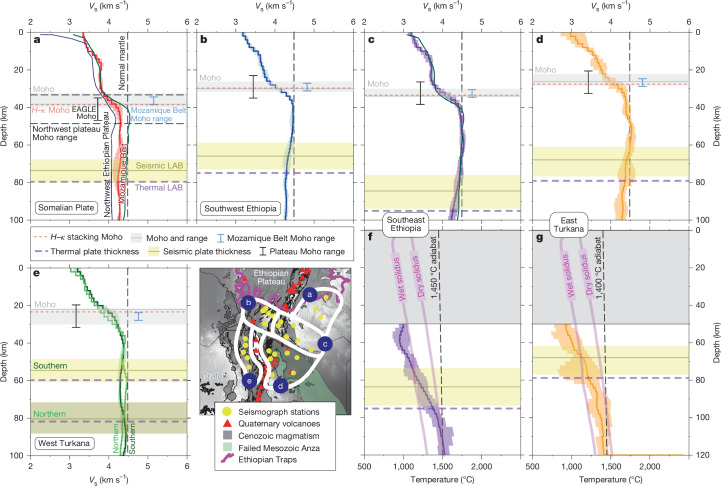
Seismic and thermal profiles across southern Ethiopia and the Turkana Depression. **a**–**e**, One-dimensional shear-wave velocity profiles grouped into domains of similar velocity: Somalian Plate (**a**), southwest Ethiopia (**b**), southeast Ethiopia (**c**), east Turkana (**d**), and west Turkana (**e**). The shading indicates the maximum velocity spread in each region. Seismically derived Moho and LAB depths, and their plausible ranges, are shown in grey and yellow, respectively. Average shear-wave profiles, and associated Moho depth ranges, for the northwest Ethiopian Plateau (dark blue line)^6^ and Mozambique Belt (dark green line)^26^ are shown in **a**. The solid black bar is the 8–12-km-thick lower-crustal-intrusion layer from the EAGLE (Ethiopia-Afar Geoscientific Lithospheric Experiment) wide-angle seismic profile on the Ethiopian Plateau^5^. The dashed red line is the *H*–*κ* stacking (H, crustal thickness; κ, *V*_P_/*V*_S_ ratio) derived Moho depths^18^. The dashed vertical line is the normal mantle velocity (4.48 km s^−1^). **f**,**g**, Temperature profiles derived from **c** and **d**, respectively, with their associated LAB depths (purple dashed line) and best-fitting mantle adiabat at asthenospheric depths (black dashed line) shown. The pink lines are the wet and dry solidi^41^. Source Data

Figure 3 shows the shear-wave velocity profiles derived from the joint inversion procedure^26^ (Methods), grouped into domains characterized by similar velocity structures at crustal and upper-mantle depths: the Somalian Plate (Fig. 3a), the broadly rifted region of southern Ethiopia (Fig. 3b,c), the failed Mesozoic Anza Rift (Fig. 3d) and the Late-Oligocene–recent rift zones west of Lake Turkana (Fig. 3e).

The Moho is identified as the base of the steepest positive velocity gradient, where typical lowermost-crustal and uppermost-mantle velocities reside (3.8–4.2 km s^−1^; Fig. 3). Defining a lithosphere–asthenosphere boundary (LAB) depth seismically is more challenging. The lithosphere remains mechanically strong to depths of about 30 km above the base of the conductive lithosphere^29^. Forward modelling suggests that the base of this predominantly conductive layer corresponds to a transition from a strong negative to a mildly positive velocity gradient^30^. Pinpointing this transition unambiguously on seismic profiles is difficult, so researchers typically estimate seismic plate thickness using the somewhat-shallower depth of the strongest negative velocity gradient. We thus assume this strategy to define a minimum LAB-depth estimate (Fig. 3).

Thermodynamic conversions of mantle shear-wave velocities to temperatures (Methods) yield temperature profiles (Fig. 3) from which a thermal proxy for LAB depth can be defined where the geotherm transitions from a conductive gradient in the uppermost mantle to an adiabatic gradient in the convective asthenosphere^30^. The temperature in the latter is no longer governed by conductive cooling and is instead unambiguously in convective mantle^30,31^. Therefore, the thermal LAB is generally deeper than its seismically derived counterpart. At asthenospheric depths, the best-fitting adiabat provides a good indication of mantle potential temperature (Fig. 3).

## Moho depth and architecture

An approximately 10-km Moho step marks the transition from thicker Ethiopian Plateau crust (about  35 km) to thinned, previously rifted, Turkana Depression crust (20–25 km; Fig. 3a–e). Crustal wavespeeds (3.2–3.8 km s^−1^) resemble Mozambique Belt terranes to the south (Fig. 3b–d) that have been un-modified by Cenozoic hotspot tectonism^26^. Unlike the Ethiopian Rift and Ethiopian Plateau (Fig. 3a), the Depression crust shows slower wavespeeds than expected for cooled gabbroic intrusions, but not slow enough to suggest high temperatures and/or melt. Our results thus corroborate previous receiver function analysis across the Depression^18^, which reveals low bulk-crustal *V*_P_/*V*_S_ ratios (about 1.74) and thin crust (<25 km) as evidence for relatively melt-poor, mechanically stretched, crust.

In addition to modifying wavespeeds, active melt and/or cooled mafic intrusions in the mid-to-lower crust are expected to fundamentally alter Moho architecture. In the heart of the Ethiopian Plateau, a substantial 8–12-km-thick lower-crustal-intrusion layer, imaged in controlled-source seismic experiments^5^, is manifest in one-dimensional velocity profiles as a gradual transition from crust to mantle velocities^6^ (Fig. 3a). In contrast, below most of the Depression, the Moho is a relatively sharp wavespeed discontinuity (<4 km; Fig. 3b–d), reminiscent of melt-poor Mozambique Belt terranes to the south^26^. Only west of Lake Turkana is a more gradational Moho found (Fig. 3e). In the north, this coincides with the Lotikipi Plain, an area of late Eocene/early Oligocene flood-basalt magmatism^32^. In the south, the Lokichar Basin contains igneous basin infill, dykes and sills^33,34^ linked to eastwards-migrating Oligocene–recent rifting from the Tanzania craton edge to its current location below Lake Turkana. Despite localized flood-basalt magmatism and Miocene–recent rifting, the Moho architecture west of the lake has been modified less than that below the Ethiopian Rift and Ethiopian Plateau (Fig. 3a). Magma-compensated rifting dominates over faulting and stretching in only the most recent (<1 Ma) extensional phase below Lake Turkana^21,35^. Recent magma-assisted rifting and/or earlier Eocene–Oligocene flood-basalt magmatism have contributed only a 2-km-thick lower-crustal-intrusion layer, imaged by the KRISP (Kenya Rift International Seismic Project) Lake Turkana wide-angle refraction profile^36^, contrasting the 8–12-km-thick layer below the Ethiopian Plateau^5^.

## Detectability of the mantle lithosphere

Melt and elevated temperatures associated with magmatic rifting and flood-basalt magmatism are expected to obscure the transition from fast-wavespeed, conductive mantle lithosphere to slow-wavespeed, convecting asthenosphere. Below the heavily melt-influenced central/northern Main Ethiopian Rift and Ethiopian Plateau, a characteristic fast-wavespeed lid is absent in one-dimensional velocity profiles^6^ (Fig. 3a); neither is a LAB ubiquitous in S-to-P receiver function studies, particularly below the Main Ethiopian Rift^37^. However, immediately to the south, southernmost Ethiopia and the Turkana Depression show a discernible, high-wavespeed mantle lithospheric lid (*V*_S_  >  4.4 km s^−1^) atop hot, slow-wavespeed (<4.3 km s^−1^) asthenosphere (Fig. 3b–d), indicating seismic and thermal lithospheric thicknesses of 65–81 km and 76–95 km, respectively, across the region (Extended Data Table 1).

A distinct lithospheric lid architecture is most prominent below the Proterozoic terranes of southern Ethiopia and the Mesozoic Anza Rift (Fig. 3c,d), where lithospheric mantle wavespeeds resemble normal mantle (about 4.48 km s^−1^). Evidence for widespread heating of the mantle lithosphere in these regions is therefore lacking, consistent with the view that Cenozoic magmatism has not heavily infiltrated or markedly modified the lithosphere to the same extent as the elevated Ethiopian Plateau. This is consistent with mantle anisotropy analysis^38^ that attributes weak anisotropy in the Depression to a paucity of melt-filled fractures in the lithosphere. Only below isolated regions of Eocene flood-basalt magmatism and Oligocene–recent rift zones west of Lake Turkana (Fig. 3e) do slower mantle wavespeeds indicate thermo-mechanical modification, albeit to a much lesser degree than the Main Ethiopian Rift and Ethiopian Plateau. Here, present-day plate thicknesses are smaller (55–60 km; Fig. 3e) compared with the rest of the Depression.

Elevated asthenospheric temperatures are ubiquitous below the Depression (Fig. 3f,g): the best-fitting mantle adiabats signify mantle potential temperatures of 1,400–1,450 °C, some 50–100 °C above ambient mantle, an observation corroborated by petrological studies of 10 Ma–recent lavas erupted near Lake Turkana^39,40^. All asthenospheric temperatures are close to, but not above, the dry solidus^41^ (Fig. 3), consistent with geochemical studies that suggest Miocene–recent magmatism below the Depression (Fig. 1b) has been pulsed, not continuous^22^. A slow-wavespeed (about 10% slower than normal mantle), hot asthenosphere, also corroborates the view that approximately 600 m of mantle-derived uplift is required to explain the Depression’s higher-than-expected elevation given its thin crust^18^. Below the Ethiopian Plateau^6^ and Somalian Plate, slow wavespeeds (<4.3 km s^−1^; Fig. 3) indicate that relatively low-density (and thus less negatively buoyant) lithospheric mantle probably contributes to their overall uplift.

Next we discuss why two adjacent regions—the Ethiopian Plateau and the Turkana Depression—that share the same geodynamic ‘plume’ setting are so profoundly different in their lithospheric structure. This contrast is particularly pronounced below the Depression’s failed Anza Rift which, despite marking a lithospheric thin spot before plume arrival, shows a surprising lack of magmatic modification.

## LIPs and plate thermo-mechanical structure

**Fig. 4:**
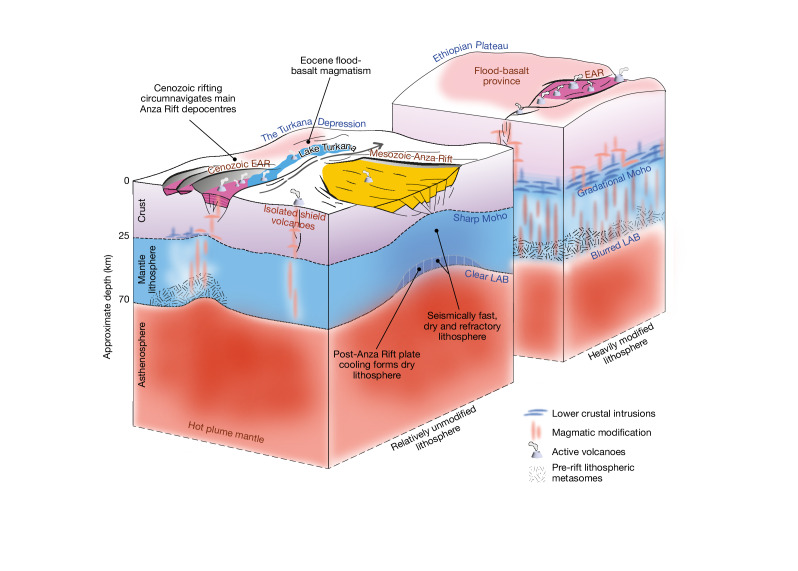
The importance of past rifting on lithospheric strength and LIP development. Schematic illustrating the key seismic and thermal observations and interpretations arising from this study. Two contrasting areas of magmatic modification above a hot, plume-influenced, mantle are shown. Thinned but relatively metasome-poor lithosphere below the failed Anza Rift is more resistant to thermo-mechanical modification than previously un-rifted regions (for example, west of Lake Turkana and the Ethiopian Plateau). EAR, East African Rift.

One hypothesis for the differences in modification is that the Depression was not underlain by a plume-affected mantle for as long as the Ethiopian Plateau. Plate reconstructions reveal that the plateau lay atop the thinnest—and therefore hottest—mantle transition zone at 30 Ma, with the Depression some approximately 500 km south of its present-day location^14^. However, this hypothesis is difficult to reconcile with the presence of some of the earliest, albeit isolated, Eocene (45–32 Ma) flood-basalt magmatism in parts of the Depression (that is, southwest Ethiopia/northwest of Lake Turkana)^4,16^. Mantle potential temperature estimates derived from Oligocene-age lavas were akin to those below the Ethiopian Plateau (+150 °C)^40^, indicating that the Depression was also underlain by hot mantle at the time. While the relative timing of plume impingement may in part explain the low-volume nature of Eocene magmatism and relatively minor levels of plate modification observed to the west of Lake Turkana (Fig. 3e), it fails to account for the lack of modification to the east, where the lithosphere was thin at the time: 3–9-km-thick Mesozoic-age extensional sedimentary basins mark the failed Anza Rift^20^. An alternative hypothesis arises from a recent modelling study^42^ that asserts that Turkana Depression lithosphere, thinned and weakened by previous rifting, inhibits melt ascent and promotes melt retention within the plate, whereas stronger, more elastic lithosphere proposed to exist below the Ethiopian Plateau readily allows magma extrusion. However, contrary to model predictions, our seismological observations find a melt-modified lithosphere associated with melt retention is absent beneath the Depression’s Anza Rift but ubiquitous below the plateau (Figs. 3 and 4).

The common assumption^2,3^ that lithospheric thin spots (for example, the Anza Rift) mark weak zones that focus deformation and facilitate the ascent of plume-related melt during the development of a flood-basalt province, does not hold true. We propose that heating and low-degree melting during short-lived magmatism associated with the late stages of Anza rifting^17,20^ would have removed easily fusible phases (for example, volatiles) from the lithosphere^43^, suppressing its potential for future thermo-mechanical modification (Fig. 4). Furthermore, partial thermal re-equilibration in the ≤20-Myr period before plume arrival and distant from any subduction zone could have rendered Anza’s newly formed lowermost mantle lithosphere relatively metasome poor. Quantifying the extent of magmatism during Anza rifting is challenging because borehole and seismic reflection data are sparse^17,20^; however, peridotite xenoliths from Marsabit Volcano (within the Anza Rift) document a history of melt extraction and depletion in incompatible element and volatile phases linked to the development of the Anza Rift, and lack evidence for metasomatic re-fertilization^44,45^. Petrological analysis of Pliocene–recent magmatism east of Lake Turkana indicates that it is predominantly asthenosphere-derived and relatively lacking in easily fusible lithospheric phases^22^ compared with Miocene lavas west of the lake^43^. This is probably owing to the more depleted nature of the Anza lithosphere: melt erupts, feeding isolated shield volcanoes, without causing widespread, seismically discernible, thermal and magmatic modification^18,27^ (Fig. 3).

Our results provide direct evidence of a fast-wavespeed and, therefore, refractory mantle lithosphere beneath the Mesozoic Anza Rift, highlighting the region’s resistance to Cenozoic plume-related modification and rifting. The Oligocene–recent geological record^20^ and analysis of present-day seismicity and geodetic data^21^ have demonstrated that East African rifting has circumnavigated, not exploited, the failed Anza Rift terranes^18,21,27^; extension instead developed to the west of Lake Turkana. Here, easily fusible phases (for example, metasomes; Fig. 4) in lithospheric mantle assembled during the Pan-African orogeny^43,46^, and steep thermal gradients at the edge of the Tanzania craton could have initiated melting with or without extension, lowering the yield strength of the lithosphere^47^, facilitating subsequent rifting and asthenospheric decompression melting. In contrast, below the failed Anza Rift, lithosphere lacking in easily fusible phases, would have been less able to undergo metasome-driven melting and associated lithospheric weakening (Fig. 4).

Although thinned lithosphere can, in some cases, enhance magmatism by promoting strain localization and drainage of decompression melt following plume arrival^1,2,9^, such as in the failed Mid-Continent Rift of North America^48^, our observations show this is not always the case. Instead we emphasize a previously underappreciated role of past rifting on the strength of the lithosphere and its susceptibility to undergo subsequent thermo-mechanical modification. Specifically, past rifting can render the mantle lithosphere dry and thus relatively stronger, not weaker, compared with neighbouring un-rifted regions. Consequently, areas of thinned lithosphere, where hot plume mantle is readily available to feed magmatism, do not necessarily develop into notable rift-related magmatic provinces, but may instead predominantly channel plume material^1,38^, facilitating modification in nearby less refractory lithosphere.

Our results have direct implications for magmatic passive margins such as the North and Central Atlantic margins^24,25,49^. Interpretations of profound along-strike variations in magmatic modification and post-rift thermal subsidence are premised on the assumption that deformation and magmatism developed preferentially in lithospheric thin spots without consideration of the physical properties of the modified lithosphere. The evidence from the Turkana Depression demonstrates that, depending on the history of deformation and melting, lithospheric thermal structure (that is, plate thickness) is not necessarily the primary controller of plate strength. The composition of the lithospheric mantle—specifically, its capacity to undergo melting in response to conductive heating alone^46^—may also be a critical factor governing magmatic rifted margin development.

## Methods

### Seismograph networks

Joint inversion was conducted on 38 temporary broadband seismograph station deployments across the Turkana Depression and southern Ethiopia (Supplementary Data Table 1). Seismograph deployments include 3 stations from the YY Ethiopian Plateau network^51^ and 34 stations from the 6R and Y1 Turkana Rift Arrays Investigating Lithospheric Structure (TRAILS) seismograph networks^52,53^. Additional data were sourced from the permanent GEOFON seismic station LODK located to the west of Lake Turkana. Seismic data were acquired through the National Science Foundation (NSF) Seismological Facility for the Advancement of Geosciences (SAGE) data archive operated by EarthScope Consortium (NSF award 1724509) and GEOFON (GEOFOrschungsNetz) repositories.

### Surface-wave dataset

Fundamental-mode Rayleigh-wave group velocity dispersion curves from a 4–60-second period for each station were extracted from the isotropic component of a local anisotropic Rayleigh-wave tomographic model^27^. Longer-period (70–100 seconds) group velocities were adopted from a global Rayleigh-wave group velocity dispersion model^28^. The local group velocity dispersion data^27^ were used for periods of 4–40 seconds or, resolution permitting, 4–60 seconds, with the final joint inversion result independent of the upper cut-off choice. To produce a final dispersion curve for each station, a three-point moving average was calculated to smooth the transition between short- and long-period group velocity data and to mitigate against sharp transitions between neighbouring periods that are otherwise not warranted given the surface-wave period–depth–sensitivity resolution kernels^27^. Collectively, these periods have peak sensitivities in a range of crustal and uppermost-mantle depths (about 5–150 km)^27^, with some partial sensitivity down to 400 km.

### Receiver function dataset

**Extended Data Fig. 1:**
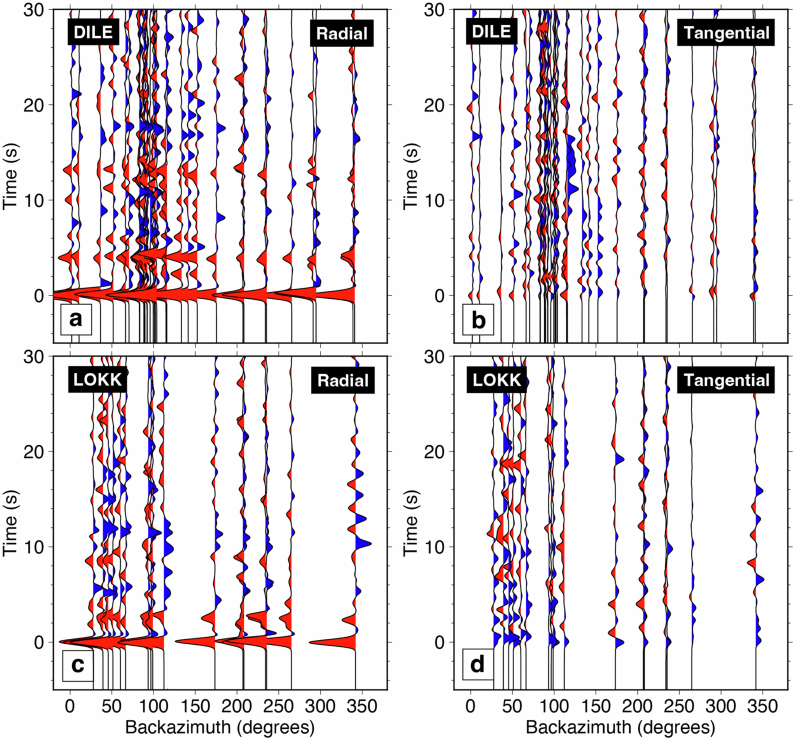
Radial and tangential receiver functions. Receiver functions with a Gaussian filter of 2.5 are plotted as a function of backazimuth for stations (**a** and **b**) DILE and (**c** and **d**) LOKK. **a** and **c**: Radial receiver functions used in the analysis. **b** and **d**: The corresponding tangential component receiver functions which were calculated to assess the impact of anisotropy.

Teleseismic earthquakes that yielded successful receiver functions were identified from a TRAILS receiver function study that used the same seismograph stations analysed here^18^. Radial P and PP receiver functions (Extended Data Fig. 1a,c) from 350 earthquakes with magnitude >4 and >5.5, respectively, were calculated using an time domain iterative deconvolution method^54^. The number of iterations are set at 250, but the deconvolution may terminate earlier if the improvement in fit is less than 0.1% for each station. For P receiver functions, earthquakes with epicentral distances of 30–90° were used, whereas for PP receiver functions, distances of ≥60° were used. Before calculating the receiver functions, seismograms were Butterworth bandpass filtered with corner frequencies of 0.04 Hz and 2 Hz. Subsequently, the seismograms were windowed from 20 seconds before to 100 seconds after the P-wave arrival, then rotated from the vertical–north–east to the vertical–radial–transverse coordinate system.

**Extended Data Fig. 2:**
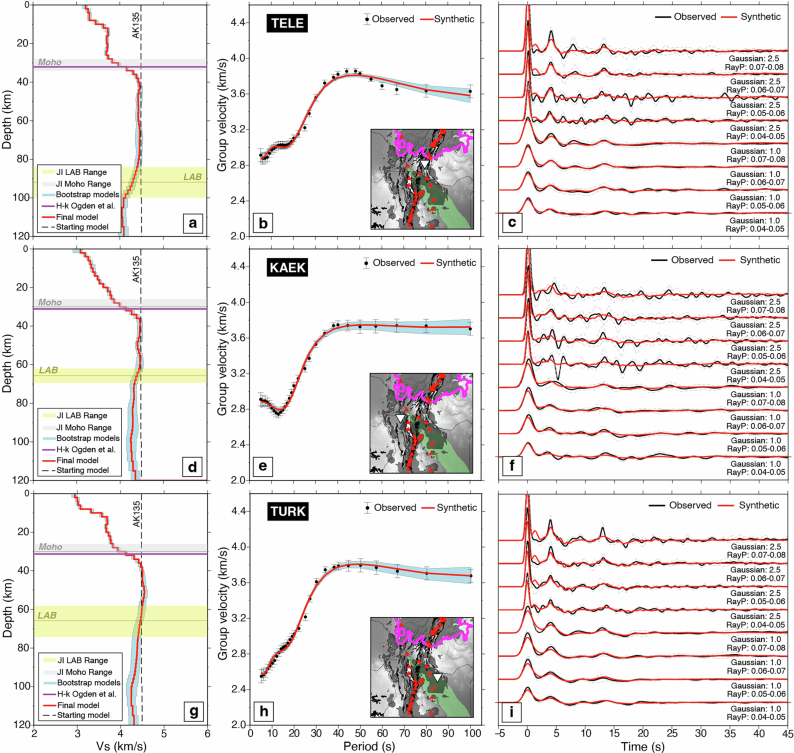
Joint inversion results for a selection of stations in southern Ethiopia and the Turkana Depression. (**a**, **d**, **g**) Final shear-wave velocity models as a function of depth (red line), including the full range of plausible bootstrap models (blue envelope) and locations/ranges of the Moho (grey line) and LAB (yellow line). The horizontal purple line is the Moho derived from H-*κ* stacking^18^. (**b**, **e**, **h**) The agreement between observed (black circles) and synthetic (red line) Rayleigh-wave group velocity dispersion data. The error bars in the observed dispersion measurements represent the mean standard deviation of the measured group velocities at each period^27,28^. (**c**, **f**, **i**) The agreement between observed (black line) and synthetic (red line) receiver functions, which are grouped by ray parameter (RayP) for two different Gaussian numbers (1.0 and 2.5). The corresponding station is shown as a white inverted triangle in the inset tectonics map, whose features are as per Fig. 1 of the main paper. Source Data

As per other receiver function^18^ and joint inversion studies performed in East Africa^6,26^, the iterative deconvolution variance—where the radial component receiver function is re-convolved with the vertical component seismogram and cross-correlated with the original radial component seismogram—was used to assess the quality of the receiver function: deconvolved traces accounting for less than 90% of the original signal were excluded from the analysis. To increase the signal-to-noise ratio of the accepted deconvolved traces, individual receiver functions for each station were stacked by taking a point-by-point average. However, variations in amplitude and timing of the phases, resulting from different incidence angles (ray parameter) of the incoming P wave can cause incoherence in stacking^26,55^. To avoid this, 4 different receiver function stacks were computed for each station by binning individual receiver functions around central ray parameter values of 0.045 s km^−1^, 0.055 s km^−1^, 0.065 s km^−1^ and 0.075 s km^−1^ (Extended Data Fig. 2c). To image details in lithospheric structure, 2 overlapping Gaussian filters (1.0 and 2.5; Extended Data Fig. 2c) were used within each of the ray parameter bins to allow for resolution of sharp versus gradational discontinuities^26,56^.

Receiver functions recorded by individual TRAILS stations were found to have high cross-correlation coefficients^18^, reflecting a lack of backazimuthal variation in structure associated with seismic anisotropy and/or heterogeneous structure (Extended Data Fig. 1a,c). Thus, additional subdivision in backazimuthal bins was not deemed necessary in this study. Tangential receiver functions were also computed to assess the potential influence from anisotropy and/or small-scale three-dimensional crustal heterogeneity^26^ below each station (Extended Data Fig. 1b,d). Although notable tangential energy is present in the melt-rich Afar Depression^57^, no notable tangential amplitudes were observed at any station below our study area (Extended Data Fig. 1b,d). An isotropic, laterally homogeneous layered structure beneath the TRAILS network and surrounding regions therefore suffices to explain the main features in the receiver function waveforms.

Of the 38 seismograph stations analysed, 26 produced reliable receiver functions. Seismograph stations located in thick sedimentary basins (for example, those nearest to Lake Turkana: BUBE, KALK, OMOE and TBIK) produced P-to-S converted energy that masked Moho arrivals, in most cases resulting in delayed receiver function P and PS phases^18,58^. Other stations situated directly on basalts which overlie 2–3-km-thick sediments (for example, in the Anza Basin: BASK, BOBE, KRGK and MAIK) also failed to produce receiver functions suitable for joint inversion^18^.

### Joint inversion procedure

Poor vertical resolution in surface waves prevents identification of sharp velocity discontinuities, such as the Moho. Surface-wave dispersion measurements are, however, more sensitive to thermal boundaries such as the LAB and readily constrain averages of absolute shear-wave velocity at different depth ranges^27^. Unlike surface-wave dispersion, receiver functions can only constrain velocity contrasts and the relative travel times for each converted phase, not absolute wavespeeds^54,56,59^. There thus exists an inherent velocity–depth trade-off. A joint inversion of surface waves and receiver functions reduces non-uniqueness in shear-wavespeed structure by ensuring the recovery of realistic shear velocities, helping mitigate trade-offs associated with each individual dataset^26,56^. Although P-to-S receiver functions mainly record crustal reverberations, which often mask reverberations from deeper discontinuities, they nevertheless also contain velocity information on deeper structure that is complementary when inverted concurrently with surface waves to recover mantle wavespeeds.

Radial P and PP receiver function stacks, within a window of −5 seconds to 45 seconds around the direct arrival, and fundamental-mode Rayleigh-wave group velocity dispersion curves for each station were jointly inverted for shear-wave velocity structure to 400 km depth using joint96^60^, an iterative least-squares inversion method^26,56^. A simple starting model was chosen to avoid including preconceived information about lithospheric structure in the inverted models—the resulting crustal and mantle lithospheric structure is thus solely dictated by the surface-wave and receiver function data. The starting model consists of 2-km-thick layers of constant *V*_S_ = 4.48 km s^−1^ (the average upper-mantle velocity of ak135)^61^ from the surface to 100 km depth. Below this, ak135 mantle velocities are used down to 400 km depth (Extended Data Fig. 2a), with layer thicknesses of 5 km between 100 km and 150 km depth and thicknesses of 10 km between 150 km and 400 km. A constant *V*_P_/*V*_S_ ratio of 1.74 was used to 40 km depth, corresponding to the average *V*_P_/*V*_S_ across all stations as determined by receiver function *H*–*κ* stacking (H, crustal thickness; κ, *V*_P_/*V*_S_ ratio)^18^. Long-period surface-wave dispersion measurements (for example, 100 seconds) have partial sensitivity to 400 km depth, meaning that deep structure can trade-off with shallower velocities if unconstrained. To minimize this trade-off, we follow common practice^6,26^ and heavily weight velocities between 300 km and 400 km to match ak135. Forward-modelled dispersion curves, derived from the final velocity model, produce a good fit to the observed long-period dispersion measurements (Extended Data Fig. 2b). Inversions were run for the number of iterations required to converge to a best-fitting model, defined as being when the misfit reduction was <0.5%.

**Extended Data Fig. 3:**
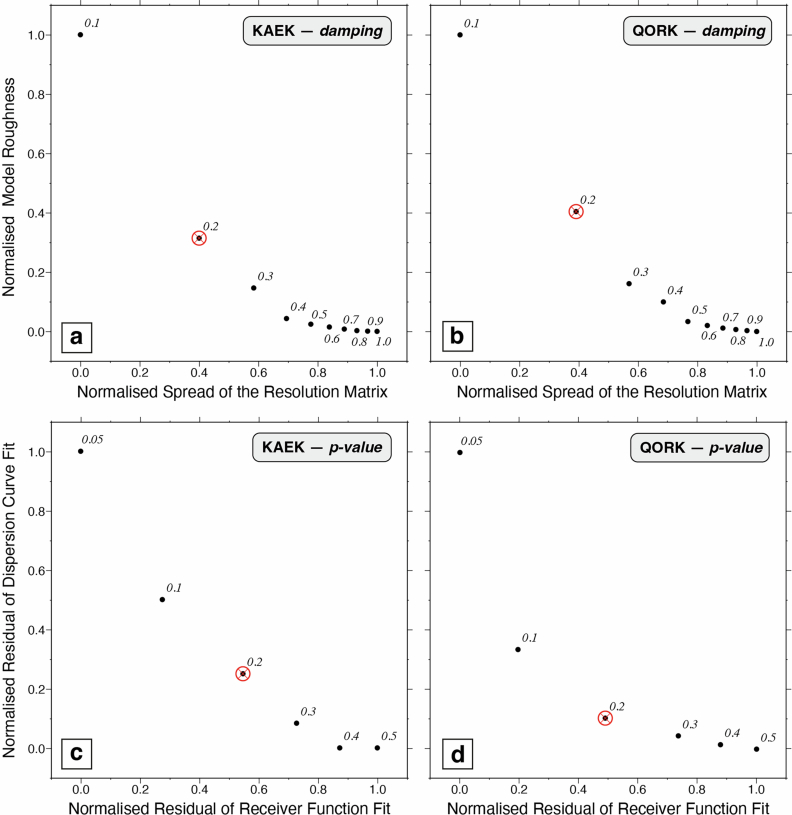
Trade-off curves for determining optimal regularization parameters. (**a** and **b**) Damping and (**c** and **d**) p-value trade-off curves for stations KAEK and QORK in the TRAILS seismograph network. The points outlined by a red circle, which lie near the ‘knee’ of the trade-off curve, denote the p-value and damping parameters used in the joint inversion of each station. Source Data

A weighting factor (*p*) controls the trade-off between fitting the receiver functions and dispersion curves; *p* = 1 indicates an inversion 100% skewed towards fitting of surface waves; *p* = 0 is a 100% receiver function inversion. A damping parameter can also be used to control the level of model smoothness and can thus influence dispersion curve and receiver function data fit. To determine the appropriate weighting and damping values, we use a trade-off curve analysis to balance model fit and complexity. First, we test a range of damping parameters (0.1–1.0) for each station while keeping *p* constant at 0.5 (that is, equal weighting between surface waves and receiver functions), and assess the trade-off between the spread of the resolution matrix and model roughness^56,62^. Damping values of 0.2 were considered optimal as they lie near the knee of the trade-off curves and are small enough to produce a good match to the receiver function waveforms for each station (Extended Data Fig. 3a,b). We subsequently, use trade-off curve analysis to determine appropriate *p* values (*p* = 0.05–0.5 were tested; Extended Data Fig. 3c,d) while keeping a constant damping value (0.2 as determined above) for each individual station in the inversion. Weighting factor values of 0.2 were chosen to maximize the contribution from receiver functions, while maintaining a good dispersion curve fit; larger *p* values (≥0.3) had negligible impact on surface-wave goodness of fit, but greatly reduced receiver function fit (Extended Data Fig. 3c,d). Differential smoothing, with smoothing levels gradually increasing with depth, was also used for all stations^56^.

Bootstrapping of receiver functions and dispersion curves for 500 iterations allowed us to assess the spread of potential models (Extended Data Fig. 2a). The dispersion curve for each station was randomly varied within minimum and maximum bounds defined by the errors in each period (Extended Data Fig. 2b). Concurrently, the selection of receiver functions that were accepted into each ray parameter stack, were also randomly varied, while allowing for duplicates. The final shear-wave velocity models are presented along with a velocity envelope signifying the spread in potential models arising from the bootstrapping process (Extended Data Fig. 2a). The corresponding spread in the synthetic dispersion curve is also shown with an envelope range (Extended Data Fig. 2b). The error in the final one-dimensional shear-wave velocity profiles at the 95% confidence level is about 0.05 km s^−1^.

Shear-wave velocity profiles for each individual station were then grouped in similar geographical domains with similar velocity profiles, where an average one-dimensional profile characterizing the region was produced (Fig. 3).

### Velocity-to-temperature conversion

To evaluate the thermal structure of the mantle beneath the Turkana Depression, we converted one-dimensional shear-wave velocities to temperature using a thermodynamic approach. Our analysis is restricted to mantle depths to avoid compositional complexities associated with the crust and Moho. The velocity-to-temperature conversion was done using phase compositions and elastic parameters computed with PerPleX^63,64^ using the stx11 database^65^ and anelasticity model Q7g^66^. Gibbs free energy minimization was used to compute phase compositions and the consequent bulk and shear moduli and density as a function of pressure and temperature, assuming a peridotitic composition. The resulting thermodynamically derived seismic velocities were corrected for anelastic effects, which depend on temperature, pressure and frequency. The correction was applied at a reference period of 1 second, which aligns with standard Earth models such as PREM and ak135, the latter of which serves as our starting model in the joint inversion. Uncertainties in the temperature estimates inherent in the thermodynamic conversion method are on the order of a few tens of degrees and are affected minimally by compositional variations, provided the composition falls within the refractory-to-fertile peridotite range^67^. The primary sources of uncertainty stem from the dependence of shear-wave velocity on resolution and inversion regularization parameters (Extended Data Fig. 3).

Temperature is expected to increase with depth within and below the lithosphere; however, the upper parts of the mantle lithosphere within some regions indicate slower wavespeeds than expected (Extended Data Fig. 2). This could perhaps be a product of radial anisotropy, which is not accounted for in the inversions, related to metasomatism in the shallow parts of the mantle lithosphere, or an artefact resulting from the smooth nature of surface-wave data and resolution kernels^68^. We thus limit temperature interpretations to deeper regions that generally follow an expected geotherm pattern, and solely for the purposes of deducing a thermal LAB estimate to corroborate our velocity proxy.

### Determining the Moho and LAB

We use the resulting one-dimensional shear-wave velocity profiles to determine Moho and LAB depths across southern Ethiopia and the Turkana Depression. The Moho is assumed to lie at the base of the steepest positive velocity gradient, where typical lowermost-crustal/uppermost-mantle velocities reside (3.8–4.2 km s^−1^). We thus calculate the slope between successive layers of the velocity profile and determine the layer with the maximum increase in velocity within this plausible Moho velocity range. These velocity limits are used to define the thickness/gradational nature of the Moho (the grey region in Fig. 3 and Extended Data Fig. 2).

A seismic proxy for the LAB is assumed to lie at the base of a high-velocity-mantle lithospheric lid, where a negative velocity gradient exists in the transition to slower-wavespeed asthenosphere. As mentioned in the main text, forward modelling suggests that the transition from conductive to convective mantle should manifest as a strong negative to mildly positive velocity gradient^29,30^; however, this is difficult to pinpoint unambiguously in seismic profiles. The depth of the strongest negative velocity gradient is often adopted as a more suitable seismic proxy for the LAB by several authors^30,69^. Before determining the LAB, we first applied a Savitzky–Golay filter^70^ to linearly smooth the velocity profiles using a sliding window of 5 km so that we can focus on long-wavelength signals and avoid the influence of small-scale heterogeneities. We then identified the window with the steepest negative velocity gradient in our smoothed velocity profiles. The gradient search was constrained to a specific depth range (≥40 km) to avoid interference from crustal gradients. The depth at which the velocity gradient first falls below the threshold of −0.01, defines the minimum (that is, the shallow limit) of our seismic LAB estimate. This gradient threshold was determined through trial and error to avoid selecting insignificant isolated anomalies in our one-dimensional profiles. A maximum LAB estimate (that is, the deeper limit) is defined by extending from the depth of the steepest negative gradient to encompass the full extent of the pronounced negative transition zone. This is achieved by mirroring the depth interval between the initial negative gradient and the steepest negative gradient. If the extrapolated depth does not coincide with a negative gradient, the algorithm adjusts by selecting the last negative gradient encountered above this depth.

**Extended Data Fig. 4:**
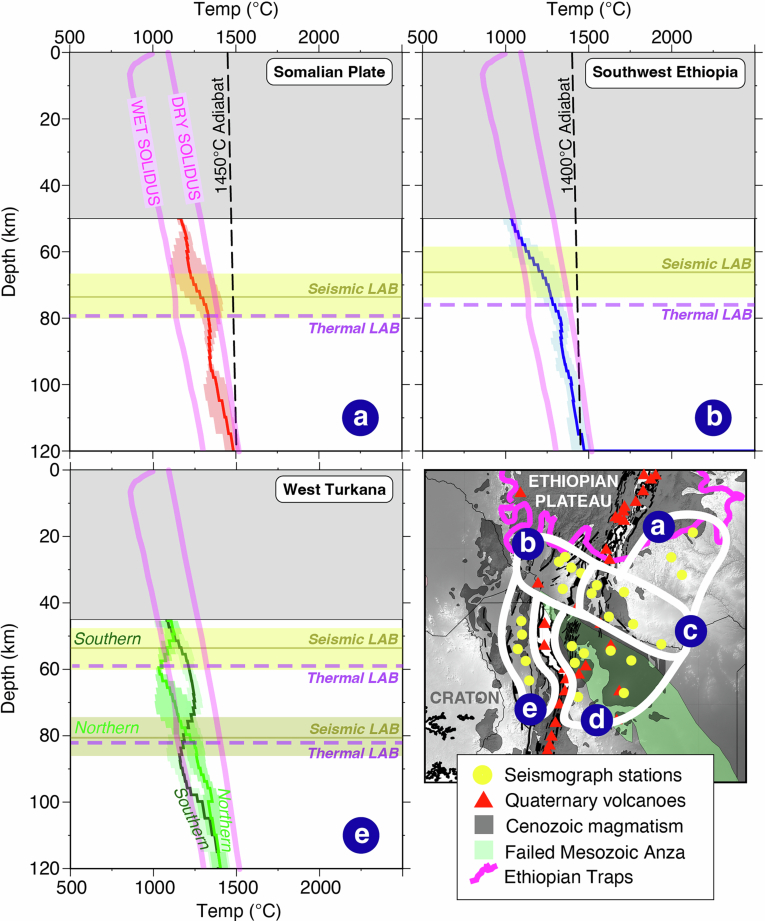
Additional thermal profiles through the Turkana Depression’s lithosphere. Averaged temperature profiles derived from regions **a**, **b** and **e**, with their associated LAB depths (purple dashed line) and best fitting mantle adiabat at asthenospheric depths (black dashed line) shown. Pink lines: the wet and dry solidus^41^.

Using a thermal proxy rather than a velocity gradient is an alternative way to infer LAB depth^30,31,69^. On the basis of our thermal profiles, we define the LAB as the depth where the geotherm shifts from a conductive gradient in the upper mantle to an adiabatic gradient in the convective asthenosphere (Fig. 3 and Extended Data Fig. 4). This is determined by manually finding the intersection point of these two gradients along the thermal profile.

## Online content

Any methods, additional references, Nature Portfolio reporting summaries, source data, extended data, supplementary information, acknowledgements, peer review information; details of author contributions and competing interests; and statements of data and code availability are available at 10.1038/s41586-025-09668-7.

## Supplementary information

Supplementary Data 1 Supplementary Data 1 contains information on the broadband seismograph stations used in our analysis, including the TRAILS network.

## Source data

Source Data Fig. 3

Source Data Extended Data Fig. 2

Source Data Extended Data Fig. 3

## Figures and Tables

**Extended Data Table 1:**
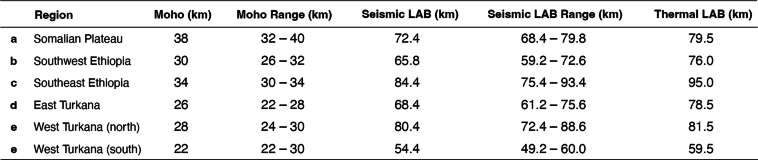
Moho and LAB depths across the Turkana Depression and southern Ethiopia

Moho and LAB depth estimates derived from our joint inversion analysis for each region defined in Fig. 3 of the main text.

## Data Availability

The facilities of EarthScope Consortium were used for access to all waveforms, related metadata and/or derived products used in this study. These services are funded through the National Science Foundation’s Seismological Facility for the Advancement of Geoscience (SAGE) Award under Cooperative Agreement EAR-1724509. All seismic data and metadata required to reproduce our analysis can be downloaded through the EarthScope Consortium Web Services (https://service.iris.edu/) for networks 6R^52^ (10.7914/SN/6R_2019), Y1^53^ (10.7914/SN/Y1_2018) and YY^51^ (10.7914/sn/yy_2013). Seismic data and metadata for network GE were obtained from the GEOFON data centre^71,72^ of the GFZ German Research Center for Geosciences (10.14470/TR560404). Source data are provided with this paper.

## Code availability

The method and codes used are standard and widely used. The joint inversion codes can be accessed via the Computer Programs in Seismology^60^ website: https://rbherrmann.github.io/ComputerProgramsSeismology/index.html. The PerPleX software^63,64^ for the temperature conversions can be accessed via this link: https://github.com/jadconnolly/Perple_X. Seismic Analysis Code (SAC)^73^ and Generic Mapping Tools (GMT)^74^ software were used to process and display seismic data.
